# Functional Testing of SLC26A4 Variants—Clinical and Molecular Analysis of a Cohort with Enlarged Vestibular Aqueduct from Austria

**DOI:** 10.3390/ijms19010209

**Published:** 2018-01-10

**Authors:** Sebastian Roesch, Emanuele Bernardinelli, Charity Nofziger, Miklós Tóth, Wolfgang Patsch, Gerd Rasp, Markus Paulmichl, Silvia Dossena

**Affiliations:** 1 Department of Otorhinolaryngology, Head and Neck Surgery, Paracelsus Medical University, Müllner Hauptstraße 48, A-5020 Salzburg, Austria; g.rasp@salk.at; 2 Institute of Pharmacology and Toxicology, Paracelsus Medical University, Strubergasse 21, A-5020 Salzburg, Austria; e.bernardinelli@pmu.ac.at (E.B.); wolfgang.patsch@pmu.ac.at (W.P.); 3 PharmGenetix Gmbh, Sonystrasse 20, A-5081 Niederalm Anif, Austria; charity.nofziger@me.com; 4 Department of Otorhinolaryngology, Head & Neck Surgery and Oncology, University Medical Center Hamburg-Eppendorf, Martinistraße 52, D-20251 Hamburg, Germany; m.toth@uke.de; 5 Center for Health and Bioresources, Austrian Institute of Technology, Muthgasse 11, A-1190 Vienna, Austria; paulmichl@me.com

**Keywords:** *SLC26A4*, non-syndromic hearing loss, enlarged vestibular aqueduct

## Abstract

The prevalence and spectrum of sequence alterations in the *SLC26A4* gene, which codes for the anion exchanger pendrin, are population-specific and account for at least 50% of cases of non-syndromic hearing loss associated with an enlarged vestibular aqueduct. A cohort of nineteen patients from Austria with hearing loss and a radiological alteration of the vestibular aqueduct underwent Sanger sequencing of *SLC26A4* and *GJB2*, coding for connexin 26. The pathogenicity of sequence alterations detected was assessed by determining ion transport and molecular features of the corresponding SLC26A4 protein variants. In this group, four uncharacterized sequence alterations within the *SLC26A4* coding region were found. Three of these lead to protein variants with abnormal functional and molecular features, while one should be considered with no pathogenic potential. Pathogenic *SLC26A4* sequence alterations were only found in 12% of patients. *SLC26A4* sequence alterations commonly found in other Caucasian populations were not detected. This survey represents the first study on the prevalence and spectrum of *SLC26A4* sequence alterations in an Austrian cohort and further suggests that genetic testing should always be integrated with functional characterization and determination of the molecular features of protein variants in order to unequivocally identify or exclude a causal link between genotype and phenotype.

## 1. Introduction

To provide the best possible care for patients affected by hearing loss, and their families, the search for a possible genetic etiology of the disease is mandatory when environmental causes are likely to be excluded. Identification of the genetic cause of deafness provides valuable information for accurate diagnosis, reliable prognosis and precise genetic counseling, and can be essential for some forms of genetic deafness that may become amenable for treatment in the future [1]. Molecular diagnostic based on sequencing of candidate genes plays a major role in this context. However, hearing loss is genetically heterogeneous and, with the exception of syndromic cases, the genotype-phenotype correlation is not straightforward. Furthermore, obtaining sufficient information on the clinical impact of a genetic variant may represent a great challenge [2].

The gap junction-β2 (*GJB2*) gene, encoding the connexin 26 protein, is the most prevalent gene associated with autosomal recessive non-syndromic hearing loss (ARNSHL) and is responsible for about 50% of cases of genetic deafness in numerous populations [3]. In addition, the causal relation between sequence alterations in the *SLC26A4* gene and hereditary hearing loss is firmly established [4,5,6,7]. Pathogenic sequence alterations in *SLC26A4* are the second most common cause of ARNSHL in most populations [1,8]. Therefore, in cases that manifest hearing loss, especially congenital sensorineural hearing loss associated with malformations of the inner ear, *SLC26A4* variants and their possible clinical implications must be considered in the molecular work-up. However, the actual pathogenic potential of many *SLC26A4* sequence alterations identified by genetic testing remains unclear due to a lack or incomplete knowledge about the function of the corresponding protein product.

The *SLC26A4* gene encodes for a protein called pendrin (SLC26A4; OMIM (Available online: http://www.ncbi.nlm.nih.gov/omim) #605646), an electroneutral Cl^−^/I^−^/HCO_3_^−^ anion exchanger [9,10,11] expressed in the inner ear [12] and thyroid [13], among other tissues. Clinical manifestations of functionally impaired SLC26A4 protein variants range from non-syndromic deafness (DFNB4; MIM #600791) to Pendred syndrome (PS; MIM #274600), the second most common type of autosomal recessive syndromic hearing loss [14]. DFNB4 is defined as hereditary hearing loss with the radiologic finding of an enlarged vestibular aqueduct (EVA), and is also referred to as non-syndromic EVA. PS is characterized by defects in the inner ear, with congenital hearing loss and EVA possibly accompanied by vestibular symptoms, as well as in the thyroid, with signs of impaired iodide organification, incompletely penetrant goiter and, occasionally, hypothyroidism [15]. The simultaneous presence of further malformations of the inner ear, in particular the Mondini malformation of the cochlea, are frequently found in the context of DFNB4 or PS [16]. The role of *SLC26A4* sequence alterations in determining PS and EVA was firmly established by several studies, either focusing on hearing impairment [17,18], alteration of the vestibular aqueduct [19], suppression of thyroid function [20] or combinations of these clinical manifestations [21]. To date, biallelic mutations of the *SLC26A4* gene are considered necessary for the development of PS, while non-syndromic EVA may be found in individuals with one, two or no *SLC26A4* mutant alleles [17,22]. The number as well as the type of *SLC26A4* mutant alleles has been found to influence the severity of hearing loss [17,18].

The spectrum and prevalence of *SLC26A4* mutations in EVA cohorts are ethnic-specific [18,23]. In this context, a literature review [24] underscores the absence of data from Austria, with the exception of isolated case reports [25]. We therefore present the analysis of the *SLC26A4* gene in a cohort of nineteen Austrian patients with hearing loss, 18 of whom have unilateral or bilateral EVA and one has bilateral agenesis of the vestibular aqueduct. Four uncharacterized *SLC26A4* variants were detected in this cohort, one of them described for the first time, and their pathogenic potential was assessed based on functional and molecular features.

## 2. Results

### 2.1. Clinical Features

Clinical information for each individual patient is summarized in Table 1, Tables S1 and S2. 

### 2.2. Detection of Sequence Variations in the GJB2 and SLC26A4 Genes

Sequence variations detected in the *GJB2* coding region and in the *SLC26A4* exons or intron-exon boundaries are summarized in Table 2. All *SLC26A4* sequence variations detected by Sanger sequencing are outlined in Table S3.

Of the four exonic *SLC26A4* sequence variations detected in this cohort, one (*c.343T>G*) is novel, for two (*c.343T>G* and *c.1301C>A*) a SNP ID was not assigned (Tables S3 and S4) and all lead to an amino acid substitution within the encoded protein. None of the four corresponding protein variants has been previously characterized on a functional or molecular level.

### 2.3. Function of SLC26A4 Protein Variants

**Figure 1:**
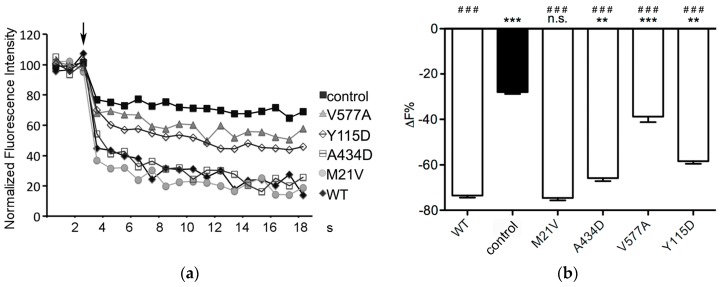
Functionality of wild type pendrin and four pendrin variants identified in Austrian deaf patients with enlarged vestibular aqueduct (EVA). (**a**) Representative measurements of intracellular fluorescence intensity in HEK 293 Phoenix cells transfected with wild type (WT) or mutated pendrin and the iodide sensor enhanced yellow fluorescent protein (EYFP) H148Q;I152L or EYFP H148Q;I152L alone (control) and bathed in chloride- or iodide-containing solutions. The arrow indicates the addition of iodide to the extracellular solution. Fluorescence intensity was normalized for the average of fluorescence intensity in the chloride-containing solution; (**b**) Percentage of fluorescence decrease (ΔF%) determined over the experimental period (19 s) in cells expressing the indicated pendrin variants and in control cells. Error bars represent SEM. *** *p* < 0.001, ** *p* < 0.01, n.s.: not statistically significant compared to wild type; ^###^ *p* < 0.001 compared to control, one-way ANOVA with Bonferroni’s multiple comparison post-test. 36 ≤ *n* ≤ 107 independent measurements collected in 3–9 independent experiments.

The function of the four pendrin protein variants (p.M21V, p.Y115D, p.A434D and p.V577A) was determined based on the ability of transporting the iodide anion following heterologous expression in HEK 293 Phoenix cells (Figure 1).

Iodide influx measured in cells expressing pendrin p.Y115D, p.A434D or p.V577A was significantly reduced compared to that measured in cells expressing wild type pendrin and significantly higher than that measured in control cells, indicating that the ion transport function of these variants, although not completely lost, is compromised. The transport activity of pendrin p.M21V was indistinguishable from wild type. The results of the functional test are summarized in Table 3.

### 2.4. Subcellular Localization of Pendrin Variants

**Figure 2:**
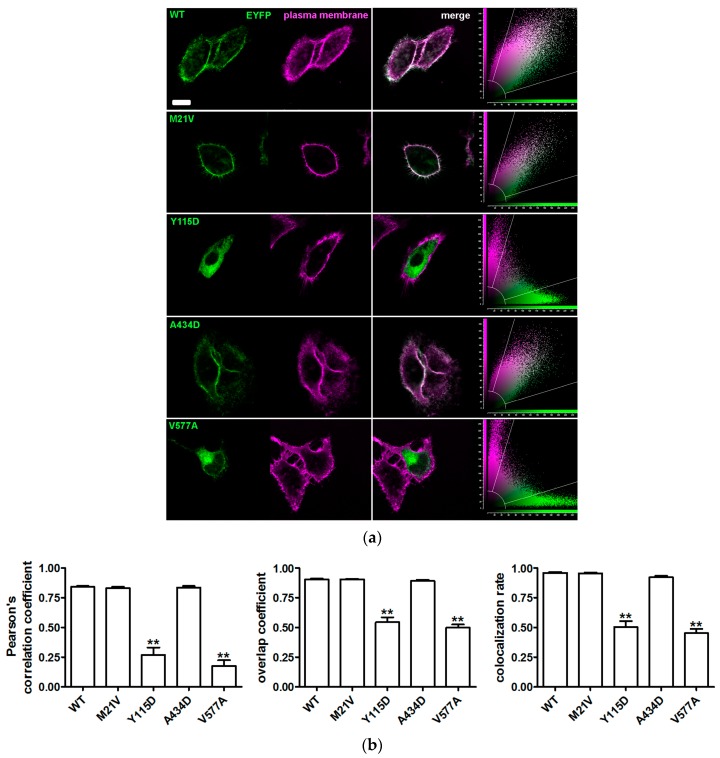
Co-localization of wild type pendrin and its variants with the plasma membrane. (**a**) From left to right: fluorescent signal of EYFP (green) fused to the C-terminus of the indicated pendrin variants and plasma membrane (magenta) of living HeLa cells 72 h after transfection, corresponding merge image and scatter plot. Scale bar: 12 µm; (**b**) Pearson’s correlation coefficient, overlap coefficient and co-localization rate referred to the co-localization of wild type (WT) pendrin and the indicated variants with the plasma membrane. Error bars represent SEM. ** *p* < 0.01 compared to wild type and therefore excluded from the plasma membrane, *n* = 12, one-way ANOVA with Dunnet’s multiple comparison post-test. (*n*) refers to the number of cells from 3 independent experiments.

**Figure 3:**
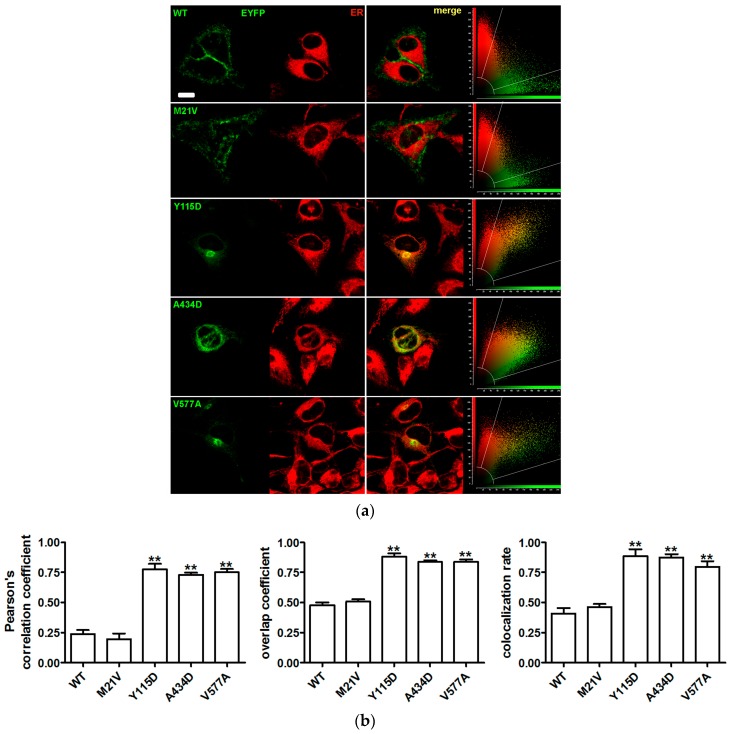
Co-localization of wild type pendrin and its variants with the endoplasmic reticulum (ER). (**a**) From left to right: fluorescent signal of EYFP (green) fused to the C-terminus of the indicated pendrin variants and ER (red) of living HeLa cells 72 h after transfection, corresponding merge image and scatter plot. Scale bar: 12 µm; (**b**) Pearson’s correlation coefficient, overlap coefficient and co-localization rate referred to the co-localization of wild type (WT) pendrin and the indicated variants with the ER. Error bars represent SEM. ** *p* < 0.01 compared to wild type and therefore retained within the ER, *n* = 12, one-way ANOVA with Dunnet’s multiple comparison post-test. (*n*) refers to the number of cells from 3 independent experiments.

To define the subcellular localization of the pendrin variants, their possible co-localization with the plasma membrane (Figure 2) or ER (Figure 3) was determined by confocal imaging following expression in HeLa cells.

Similarly to wild type pendrin, pendrin p.M21V co-localized with the plasma membrane and not with the ER. Conversely, pendrin p.Y115D and p.V577A did not reach the plasma membrane, but did co-localize with the ER. Pendrin p.A434D co-localized with both the plasma membrane and ER. The results of the subcellular localization studies are summarized in Table 3.

### 2.5. Total and Plasma Membrane Expression Levels of Pendrin Variants

**Figure 4:**
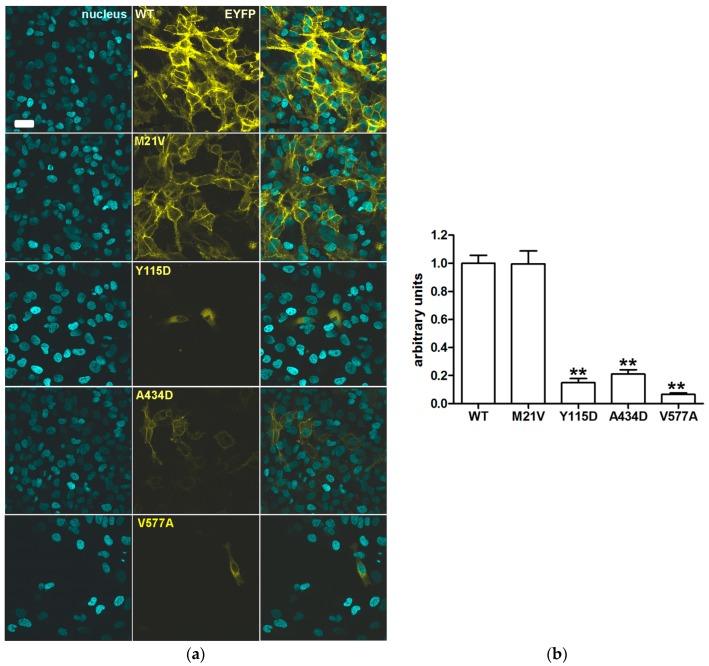
Total cellular levels of wild type pendrin and its variants in intact cells. (**a**) From left to right: nuclei counterstained with 4’,6-diamidino-2-phenylindole (DAPI, cyan), fluorescent signal of EYFP (yellow) fused to the C-terminus of the indicated pendrin variants expressed in HeLa cells for 72 h and corresponding merge image. Scale bar: 30 µm; (**b**) Wild type (WT) and mutated pendrin total expression levels expressed as fluorescence intensity (levels of grey) normalized for the cell density. Error bars represent SEM. *n* = 24, ** *p* < 0.01 compared to wild type, one-way ANOVA with Dunnet’s multiple comparison post-test. (*n*) refers to the number of imaging fields from 6 independent experiments.

Expression levels of pendrin variants were determined in the whole cell and in the region of the plasma membrane by confocal imaging following expression in HeLa cells. The total expression levels of all of the functionally impaired pendrin variants (p.Y115D, p.A434D and p.V577A) were substantially reduced with respect to those of the wild type. In contrast, the total expression levels of the fully functional pendrin variant p.M21V were not significantly different from the wild type (Figure 4).

**Figure 5:**
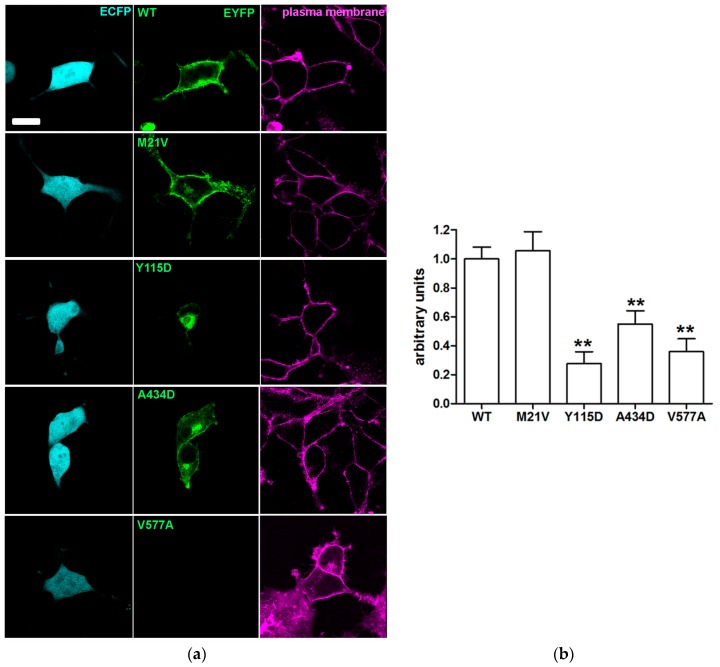
Abundance of wild type pendrin and its variants in the plasma membrane region of living cells. (**a**) From left to right: fluorescent signal of the transfection marker enhanced cyan fluorescent protein (ECFP, cyan), EYFP fused to the C-terminus of the indicated pendrin variants expressed in HeLa cells for 72 h (green), and plasma membrane (magenta). Scale bar: 15 µm; (**b**) Wild type (WT) and mutated pendrin fluorescence intensity in three regions of interest of the plasma membrane of a single cell were expressed as levels of grey, averaged and normalized for the fluorescence intensity of ECFP in the cytosol of the same cell. Error bars represent SEM. *n* = 24, ** *p* < 0.01 compared to wild type, one-way ANOVA with Bonferroni’s multiple comparison post-test. (*n*) refers to the number cells from 6 independent experiments.

Consistent with previous observations [27], expression levels of pendrin variants in the plasma membrane region determined on a single-cell level (Figure 5) mirrored total expression levels. Expression levels of pendrin variants are summarized in Table 3.

### 2.6. Population Frequencies of c.343T>G and c.1301C>A

**Figure 6:**
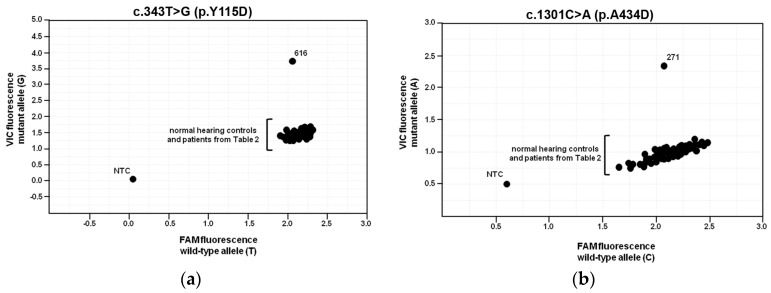
Allelic discrimination plots from custom genotyping assays. The original scatter plots generated from the custom genotyping assays for *c.343T>G* (p.Y115D) (**a**) and *c.1301C>A* (p.A434D) (**b**). Little to no fluorescence from either fluorophore in either assay was generated in the no template controls (NTC). Raw endpoint fluorescence data were analyzed with QuantStudio 12K Flex software v1.2.2 and scatter plots were generated with Taqman Genotyping software v1.3 (Thermo Fisher Scientific, Waltham, MA, USA).

The presence of the two (*c.343T>G* and *c.1301C>A*) exonic *SLC26A4* sequence variations was assessed in 125 healthy volunteers with no self-reported hearing disorders using custom designed genotyping assays. The genotyping results for patient 616 (heterozygous for *c.343T>G*) and patient 271 (heterozygous for *c.1301C>A*) and all other patients outlined in Table 2 (wild-type at both positions) matched that from the Sanger sequencing results, indicating that both custom genotyping assays worked correctly (Figure 6). All of the normal hearing control samples were homozygous wild-type for both sequence variations. The frequency of both sequence variants was 0.69% and each fulfilled the Hardy-Weinberg equilibrium expectation. 

## 3. Discussion

A great diversity of clinical manifestations, in particular audiometric characteristics of hearing loss and vestibular symptoms, typically reported among patients with EVA [28,29,30], was confirmed in our cohort of 19 patients from Austria, including one patient with agenesis of the vestibular aqueduct on both sides (Table 1, Tables S1 and S2). An intraoperative gusher phenomenon of cerebro-spinal fluid during cochlea implant surgery was observed in 7 of 23 operated ears (30.4%) (Table S1), an incidence that is relatively high compared to other groups [31,32]. However, the condition was manageable in all cases by accurate sealing during surgery without any postoperative leakage observed. The incidence was independent from *SLC26A4* genotype.

A total of 12 of 19 patients (63.2%), including the patient with agenesis of the vestibular aqueduct, complained of dizziness or vertigo to a certain extent (Table S2). This is in agreement with other reports [29,33]. However, Zalewski et al. [29] concluded that, although vestibular symptoms are commonly found in EVA patients, a causative relation cannot be identified. Vestibular disorders, such as Menière’s disease or vestibular migraine, need to be considered in cases of reported episodic or progressive dizziness or vertigo. Clinical diagnosis of these vestibular disorders is based on clinical findings defined by Lopez-Escamez et al. [34] for Menière’s disease and Lempert et al. [35] for vestibular migraine. None of our patients fulfilled the diagnostic criteria for any of those two diagnoses. None of the patients had a low frequency sensorineural hearing loss. Reported vestibular symptoms did not appear in repeated attacks accompanied by cochlear symptoms, such as tinnitus or aural fullness. Only one patient (patient ID 395) reported headache but not in connection with episodic vertigo and without any sensation of an aura (Table S2). Caloric testing and head impulse testing results are reported in Table S2. Vestibular diagnosis in patients undergoing cochlear implant at our department is done prior to surgery in patients older than 15 years. Therefore, data on vestibular function are not available for all patients. Unfortunately, there is no objective data on vestibular function of patient 271 (bearing biallelic sequence alterations in *SLC26A4*) due to young age. However, parents did not report difficulties in motor development, giving a clinical hint on adequate vestibular function. Patient 358 reported episodic dizziness and showed symmetric reduction of excitability during caloric testing on both sides. Patient 616 did not show signs of vestibular dysfunction with physiological caloric testing and head impulse test.

Two previously reported *GJB2* variants (*c.35delG* and *c.88A>G*) were found in our cohort (Table 2). The *GJB2* variant *c.35delG*, leading to premature protein chain termination, was unequivocally established by numerous studies as pathogenic [36,37] and represents the most common cause of hearing loss worldwide [24]. In our cohort, the variant *c.35delG* was detected in homozygosity in two unrelated Caucasian patients with bilateral EVA and no *SLC26A4* sequence alterations, and can reasonably be considered as the genetic determinant of deafness in these individuals (Table 2). 

The *GJB2* variant *c.88A>G* leads to the amino acid substitution p.I30V, and was first detected as a single allelic mutation in one patient of a cohort of 324 Taiwanese individuals with prelingual deafness [38]. To our knowledge, no functional or molecular tests for the GJB2 variant p.I30V have been performed. However, bioinformatic predictions indicate that this amino acid change may be tolerated, and the corresponding protein variant may have no pathogenic potential [39]. In our cohort, the *c.88A>G GJB2* variant was found in two siblings of Arab descent (patient ID 267 and 308, Table 1 and Table 2) with bilateral EVA, renal tubular acidosis (RTA) and no sequence alterations on the other *GJB2* allele. Whether monoallelic *GJB2* sequence alterations can be sufficient to determine deafness is currently unclear [37,40]. Importantly, mutations in *ATP6V1B1*, which encodes the B1-subunit of H^+^-ATPase, can cause RTA, early onset hearing loss and bilateral EVA [41] and may reasonably account for the pathological phenotype encountered in these two subjects. 

Although the link between *GJB2* mutations and temporal bone anomalies is not clearly established [42,43], *GJB2* variants can be detected in EVA patients with variable prevalence depending on the specific population and the criterion adopted to define EVA [43,44,45]. In our cohort, both patients with biallelic *GJB2* mutations (patient ID 307 and 421) presented bilateral EVA, thus supporting a link between *GJB2* dysfunction and inner ear malformations. It is possible, however, that the homozygous variant *c.35delG* determines non-syndromic hearing loss in these patients, which also manifest EVA as the consequence of undetected pathogenic sequence alterations in *SLC26A4* or other genes. Of note, both patients show signs of vestibular dysfunction (Table S2) and patient 307 presents the most severe hearing loss phenotype (bilateral complete absence of hearing function) observed in this cohort (Table S1).

Four *SLC26A4* variants were found in this cohort (Table 2); for two of them (*c.61A>G*, SNP ID rs375716219, and *c.1730T>C*, SNP ID rs56017519) an SNP ID was assigned, however, functional characterization is missing (according to: https://www.ncbi.nlm.nih.gov/snp/, the clinical significance was not assessed for the former and uncertain for the latter, respectively). The other two *SLC26A4* variants (*c.343T>G* and *c.1301C>A*) are not reported in the SNP database. To assess their pathogenic potential, the functional and molecular features of the corresponding protein variants were assessed by in vitro studies, and the protein variants were subsequently classified according to the American College of Medical Genetics and Genomics Standards and Guidelines [26] (Table 3).

Sequence alterations *c.1301C>A* and *c.1730T>C* encoding the pendrin protein variants p.A434D and p.V577A were found in compound heterozygosity in a patient with congenital hearing loss, incomplete partition 2 (IP2) and no sequence alterations in *GJB2* (patient ID 271, Table 1 and Table 2). The former sequence alteration was inherited from the mother and the latter from the father, both of whom had normal hearing. Variant p.A434D showed a 16% reduction of iodide transport activity (Figure 1 and Table 3). Following heterologous expression, this variant co-localized with both the plasma membrane (Figure 2) and the ER (Figure 3). The total cellular and plasma membrane expression levels of this variant appear substantially reduced compared to the wild type (Figure 4 and Figure 5), although residual expression (*p* < 0.001 compared to p.V577A, Figure 4) and partial plasma membrane targeting (Figure 2) allowed for residual transport activity (Figure 1). The functional and molecular defects of variant p.V577A appeared more severe in terms of loss in ion transport function (76% reduction, Figure 1 and Table 3), complete retention in the ER (Figure 2 and Figure 3) and dramatic reduction of expression (Figure 4 and Figure 5). We conclude that both SLC26A4 variants show functional and molecular features compatible with the pathological phenotype of the patient (Table 3). Evidence of thyroid malfunction due to pendrin dysfunction may unequivocally be obtained through perchlorate discharge testing [22]. Assessing the thyroid function by perchlorate testing in this patient was not possible; therefore, definite discrimination between non-syndromic EVA and Pendred syndrome could not be ascertained. Ultrasound of the thyroid gland showed no signs of pathologic volume changes. To date, the patient is eight years old, and manifest goiter may appear in the second decade of life [46,47]. A clinical follow-up will be performed including perchlorate testing when possible.

The *SLC26A4* variant *c.343T>G* was found in heterozygosity with the wild type allele in an individual with fluctuating hearing loss, bilateral EVA and no *GJB2* mutations (patient ID 616, Table 1 and Table 2). The corresponding protein variant p.Y115D showed substantially reduced activity and expression levels (Figure 1, Figure 4 and Figure 5 and Table 3), loss of targeting at the plasma membrane and retention within the ER (Figure 2 and Figure 3). The functional and molecular features of this variant are consistent with a pathogenic potential when another dysfunctional allele is present (Table 3). Whether monoallelic pendrin mutations are sufficient to determine a pathological phenotype is currently unclear. Some studies support the hypothesis of a second, undetected *SLC26A4* mutation that accounts for EVA in these cases [48,49]. For example, sequence alterations within the promoter or cis-regulatory (enhancer) regions leading to a reduced transcription may per se be insufficient to determine a pathological phenotype, but can be pathogenic in combination with another dysfunctional allele. These sequence alterations may reside in upstream regions distant from *SLC26A4*, intergenic regions or even other genes [50]. For this patient, the only other sequence alteration in *SLC26A4* with an allele frequency <1%, of which the clinical significance was not assessed, was found in the 3’UTR (g.55910G>A, rs375630290, Table S3). Therefore, the genetic cause of deafness for this patient remains undetermined. 

For 7 of 19 patients (6 of 18 families, 33%) a family history of hearing loss was recorded (Table S1). The pedigree of these families is presented in Figure S1. No candidate causative variants were identified for these familial cases (as discussed above, the *GJB2* variant c.88A>G found in patients 267 and 308 and the *SLC26A4* variant c.61A>G found in patient 358 are most likely with no pathogenic potential). 

PS/non-syndromic EVA have also been associated with monoallelic sequence alterations in the *SLC26A4* and *FOXI1* or *KCNJ10* genes, with a double-heterozygosity inheritance model [51,52]. FOXI1 (OMIM #601093) is a transcription factor for *SLC26A4* [52,53], and KCNJ10 (OMIM #602208) is an inwardly rectifying K^+^ channel essential for the maintenance of the endocochlear potential [54]. Although later studies showed that sequence alterations in *FOXI1* or *KCNJ10* are rare in the context of PS/non-syndromic EVA [55,56,57,58], analysis of these genes may be of value in individuals with monoallelic pathogenic *SLC26A4* sequence alterations.

SLC26A4 variants with no reduction in ion transport function are found in the general [59,60], deaf with no EVA [59,60] and deaf with EVA [27,61,62,63,64] populations. Including these variants into genotype-phenotype correlation analyses may lead to false conclusions [65]. Therefore, unequivocal assessment of pathogenic potential of a *SLC26A4* sequence alteration requires functional studies of the corresponding protein variant. In our cohort, the *SLC26A4* variant *c.61A>G* was found in heterozygosity with the wild type allele in an individual with profound hearing loss, bilateral IP2 and no *GJB2* mutations (patient ID 358, Table 1 and Table 2). The corresponding protein variant p.M21V did not show alterations in function (Figure 1), subcellular localization (Figure 2 and Figure 3) or expression levels (Figure 4 and Figure 5) compared to the wild type, and should therefore be considered as having no pathogenic potential (Table 3). No additional sequence alterations with an allele frequency <1% were found in *SLC26A4* in this patient (Table S3), therefore pendrin dysfunction most likely does not account for the hearing loss in this patient.

In Caucasian cohorts, approximately 50% of all individuals with unilateral or bilateral hearing loss and EVA carry at least one mutant *SLC26A4* allele [22,23,66]. For example, in a cohort of 100 unrelated French individuals with EVA, *SLC26A4* mutations (either monoallelic or biallelic) were found in 40% of subjects [67]. In our cohort of 17 unrelated individuals with EVA, only 2 (12%) presented at least one pathogenic *SLC26A4* allele (*p* = 0.02 compared to the above mentioned French cohort). Therefore, *SLC26A4* mutations seem to be under-represented in our cohort, thus indicating that additional genes may be involved in determining EVA in the Austrian population. 

The spectrum of *SLC26A4* variants is strictly related to the ethnicity of patients. The *c.1001+1G>A*, p.V138F, p.T416P, p.L236P and p.G209V pathogenic variants are prevalent in the Caucasian population, with p.V138F being predominant in Germany and Czech Republic, two countries geographically close to Austria [24]. None of these mutations were found in our cohort. Indeed, a novel pathogenic variant (p.Y115D) was detected. The novel pathogenic variant was not detected in 125 population-matched healthy control individuals (Figure 6). In addition, the previously reported *SLC26A4* variants found in our cohort (p.M21V and p.V577A) are not frequently found in the general population—according to: https://www.ncbi.nlm.nih.gov/snp/, the minor allele frequency (MAF) is 0.0042% for *c.61A>G* (p.M21V), SNP ID: rs375716219, and 0.001647% for *c.1730T>C* (p.V577A), SNP ID: rs56017519, respectively (Table S4)—nor in deaf individuals [24]. The MAF of all four exonic *SLC26A4* variants (Table S4) is lower than 0.005, a threshold value for pathogenic autosomal-recessive variants suggested by Shearer et al. [68]. These observations underscore that *SLC26A4* variants found in our cohort, including the variant characterized as non-pathogenic in this work, are uncommon in the general population. Therefore, in terms of prevalence and spectrum of *SLC26A4* mutations, the Austrian population seems to present characteristics distinct from those of other Caucasian-European groups. Expansion of the cohort subject of this study will be essential to establish if the pathogenic *SLC26A4* allelic variants more commonly found in Caucasians are either rare or absent in the Austrian population. 

## 4. Materials and Methods 

### 4.1. Patients

Eighteen Austrian subjects (10 females and 8 males aged between 8 and 51 years; average age 27 years) diagnosed with sensorineural (*n* = 16), mixed (*n* = 1) or conductive (*n* = 1) hearing loss and EVA, as well as one subject with agenesis of the vestibular aqueduct (patient ID 365) were included in the study (Table 1). Of these, two (patient ID 267 and 308) were siblings of Arab origin. All the other patients were unrelated subjects of Caucasian, or mixed Caucasian and African (patient ID 395) ethnicity. The research was prospectively reviewed and approved by a duly constituted ethics committee (415-E/2092/6-2017, 9 May 2017) and has therefore been performed in accordance with the principles embodied in the 1964 Declaration of Helsinki and its later amendments (Available online: https://www.wma.net/policies-post/wma-declaration-of-helsinki-ethical-principles-for-medical-research-involving-human-subjects/). Written informed consent was obtained from all subjects or their legal representatives prior to blood sampling and genetic testing. For all patients, imaging studies of the inner ear by computer tomography (CT) of the temporal bones were performed. EVA was defined as an enlargement of the vestibular aqueduct ≥1.5 mm midway between the endolymphatic sac and the vestibule. An abnormal cochlea was considered as Mondini malformation in cases of a normal basal turn and cavity-like appearing distal turns with a missing interscalar ridge between the basal turn and the distal turns on the axial plane of the CT scan [69]. IP2 was defined as the combination of EVA and Mondini malformation [70]. None of the patients presented overt goiter or alterations of clinical or laboratory thyroid functional parameters. Individual characterization of hearing loss (HL) was based on side-specific pure-tone audiometric testing results, as well as the patient’s history. The type of HL was defined as conductive, mixed or sensorineural based on air- and bone-conduction thresholds. The severity of HL was classified on decibel hearing level (dB HL) thresholds, either as mild for values between 26 and 40 dB HL, moderate between 41 and 60 dB HL, severe between 61 and 80 dB HL or profound for values above 80 dB HL. Ears with no perception at all were considered as deaf. Frequencies possibly affected by HL were divided into low, middle or high frequency range. Onset of HL was defined through patient’s history, since newborn screening tests were either not performed or results were not available. Related to the time of speech development, the onset of HL was defined as congenital, prelingual, perilingual or postlingual. Clinical course of HL with possible hearing drops was obtained through audiometric tests and patient’s history. Surgical reports were screened for intraoperative gusher phenomenon, defined as visible efflux of cerebro-spinal fluid after cochleostomy, during cochlear implant surgery.

### 4.2. Genomic DNA Samples

Patient whole blood was collected in plastic tubes with potassium-ethylenediaminetetraacetic acid (S-Monovette^®^, Sarstedt, Nümbrecht, Germany) via venipuncture. Total genomic DNA (gDNA) was purified from ~350 μL blood with the EZ1 DNA Blood 350 μL kit (Qiagen, Hilden, Germany) using the EZ1 Advanced XL platform (Qiagen) according to the manufacturer’s instructions. Quantification was performed with the QIAxpert (Qiagen) spectrophotometer. Only samples with an A260/A280 between 1.7 and 1.9 were used for downstream analysis.

### 4.3. Genomic DNA Analysis

Fifty μL endpoint polymerase chain reaction (PCR) reactions for amplifying different areas of the *SLC26A4* gene contained 1× JumpStart REDAccuTaq Long and Accurate (JS RAT LA) DNA Polymerase buffer (Sigma-Aldrich, St. Louis, MO, USA), 1 mM dNTPs (Thermo Fisher Scientific; Waltham, MA, USA), 10% dimethyl sulfoxide (Sigma-Aldrich), 0.8 μM forward and reverse primers and 5 units JS RAT LA DNA Polymerase (Sigma-Aldrich). Amplification and sequencing primers and cycling parameters are described in Table S5.

The entire coding region of *GJB2* (exon 2) was amplified by PCR as described above using the following primers: forward, 5′ CACGTTCAAGAGGGTTTG 3′, and reverse, 5′ TGAGCCTTGACAGCTGAGC 3′. The same primers were used for sequencing.

The PCR products were purified and Sanger sequenced (Microsynth AG, Balgach, Switzerland). The resulting sequences were compared against the NCBI *Homo sapiens GJB2* (OMIM ID: 121011, GenBank ID: NG_008358.1) or *SLC26A4* (OMIM ID: 605646, NCBI ID: AC078937.1) DNA sequence reference assembly. 

### 4.4. Plasmid Constructs

The pTARGET (Promega Corporation, Madison, WI, USA) vector, containing the cDNA of wild type or mutated human pendrin [9], was used for functional tests. 

The pEYFPN1 vector (Clontech, Mountain View, CA, USA), containing the cDNA of wild type or mutated human pendrin, was used for co-localization and determination of pendrin expression levels via imaging. Following transfection of this construct in cells, pendrin is produced with the enhanced yellow fluorescent protein (EYFP) fused to its C-terminus [27]. 

Sequence alterations in the cDNA of pendrin were obtained using the QuikChange^®^ site-directed mutagenesis kit (Stratagene, La Jolla, CA, USA) and the primers listed in the Table S6.

The sequence of all plasmid inserts was verified by Sanger sequencing (Microsynth AG).

### 4.5. Cell Lines 

Human embryonic kidney (HEK) 293 Phoenix [71] and HeLa (cervical adenocarcinoma, CCL-2, American Type Cell Culture Collection, Manassas, VA, USA) cells were cultured as previously described [9,27].

### 4.6. Pendrin Functional Test 

For testing the function of pendrin variants, the influx of iodide was measured in cells expressing wild type or mutated pendrin and in control cells. The functional test was performed as already described [9,59,72,73], with adaptations allowing for the use of a multiplate reader [27,60,74,75,76]. Shortly, cells were seeded into black 96-well plates, grown overnight and co-transfected with 0.1 μg of a plasmid encoding for EYFP H148Q;I152L (an EYFP variant with substantially improved sensitivity for iodide [77]) and 0.1 μg of pTARGET plasmid bearing the cDNA of wild type or mutated pendrin. Control cells were co-transfected with EYFP H148Q;I152L and the empty pTARGET vectors. Transfection was done by the calcium phosphate co-precipitation method. Experiments were performed at room temperature 48 h post-transfection. Cells were initially bathed in 70 μL of high chloride solution (in mM: KCl 2, NaCl 135, CaCl_2_ 1, MgCl_2_ 1, d-glucose 10, 4-(2-hydroxyethyl)-1-piperazineethane sulfonic acid (HEPES) 20, 308 mOsm/KgH_2_O with mannitol, pH 7.4), and the baseline fluorescence intensity was measured (1 measurement/s for 3 s). Subsequently, 140 μL of high iodide solution (in mM: KCl 2, NaI 135, CaCl_2_ 1, MgCl_2_ 1, d-glucose 10, HEPES 20, 308 mOsm/KgH_2_O with mannitol, pH 7.4) were injected into each well and the fluorescence intensity was measured again (1 measurement/s for 16 s). Owing on the chloride/iodide exchanger activity of pendrin [9,10,11], iodide enters the cytosol of pendrin-expressing cells from the extracellular medium and leads to a decrease of EYFP H148Q;I152L fluorescence. Therefore, negative % fluorescence variations (ΔF%) indicate a flux of iodide from the extracellular to the intracellular milieu and reflect pendrin transport efficiency.

Fluorescence intensity was quantified with the VICTOR^TM^ X3 Multilabel Plate Reader (Perkin Elmer, Waltham, MA, USA) equipped with a liquid dispenser and the following filters: excitation: F485 (excitation center wavelength (CWL): 485 nm, bandwidth: 14 nm), emission: F535 (emission CWL: 535 nm, bandwidth: 25 nm). 

### 4.7. Co-Localization Experiments

HeLa cells were seeded into 6-well plates, grown overnight, transfected with 1.5 μg of plasmid DNA and 3 µL of Metafectene Pro (Biontex, München, Germany), transferred on glass slides for microscopy (48 h post-transfection) and imaged (72 h post-transfection).

Subcellular localization of pendrin variants was determined by co-localization between wild type or mutant SLC26A4 with EYFP fused to the C-terminus and markers of the plasma membrane (CellMask^TM^ Deep Red plasma membrane stain, C10046, Invitrogen Molecular Probes, Waltham, MA, USA) or endoplasmic reticulum (ER) (ER-Tracker^TM^ Red, BODIPY^TM^ TR glibenclamide, E34250, Invitrogen Molecular Probes). 

To stain the plasma membrane, living cells were washed thrice with ice-cold Hank’s balanced salt solution (HBSS, Sigma-Aldrich), incubated on ice for 5 min with 1.25 µg/mL CellMask^TM^ Deep Red plasma membrane stain in HBSS, washed again thrice with ice-cold HBSS and imaged immediately in HBSS. 

To stain the ER, living cells were washed thrice with room temperature HBSS, incubated for 20 min at 37 °C and 5% CO_2_ with 1 µM ER-Tracker^TM^ Red in Krebs-Henseleit buffer (Sigma-Aldrich), washed again thrice and immediately imaged in HBSS at room temperature.

Co-localization was detected and quantified as previously described [27]. Shortly, imaging was performed by sequential acquisition with a Leica TCS SP5II AOBS confocal microscope (Leica Microsystems, Wetzlar, Germany) equipped with a HCX PL APO 63×/1.20 Lambda blue water immersion objective and controlled by the LAS AF SP5 software (Leica Microsystems). For co-localization with the plasma membrane, EYFP was excited with the 514 nm line of the Argon laser and emission was detected in the 525–600 nm range; the CellMask^TM^ Deep Red stain was excited at 633 nm (Helium Neon laser) and emission was detected in the 655–750 nm range. For co-localization with the ER, EYFP was excited with the 514 nm line of the Argon laser and emission was detected in the 525–555 nm range; ER-Tracker^TM^ Red was excited at 561 nm (diode-pumped solid-state (DPSS) laser) and emission was detected in the 571–650 nm range. Co-localization with plasma membrane or ER markers was quantified and expressed as the Pearson’s correlation coefficient [78], overlap coefficient and co-localization rate. The co-localization parameters of wild type pendrin were considered as indicators of a preferential co-localization with the plasma membrane and poor co-localization with the ER.

### 4.8. Determination of Wild Type and Mutant Pendrin Total Expression Levels by Imaging

Determination of total expression levels of pendrin variants was performed as previously described [27]. Shortly, HeLa cells expressing wild type or mutant SLC26A4 with EYFP fused to the C-terminus were fixed with 4% paraformaldehyde for 30 min, counterstained with 0.1 μg/mL 4′,6-diamidino-2-phenylindole (DAPI) for 10 min, washed and imaged in HBSS. Wild type or mutant SLC26A4 expression levels, given by the fluorescence intensity of EYFP, were normalized for the cell density, given by the fluorescence intensity of DAPI. Imaging was performed by confocal microscopy as described above. EYFP was excited with the 514 nm line of the Argon laser and emission was detected between 525 and 600 nm; DAPI was excited with a diode laser (405 nm) and emission was detected between 430 and 470 nm. Laser power and photomultipliers gain were kept rigorously constant for acquisition of all images.

### 4.9. Determination of Wild Type and Mutant Pendrin Expression Levels in the Plasma Membrane Region

Determination of expression levels of pendrin variants in the region of the plasma membrane was performed as previously described [27]. Shortly, HeLa cells expressing wild type or mutant SLC26A4 with EYFP fused to the C-terminus and the enhanced cyan fluorescent protein (ECFP) (pECFPC1 vector, Clontech) were stained with CellMask^TM^ Deep Red plasma membrane stain, washed and imaged in HBSS. EYFP fluorescence intensity of three regions of interest of the plasma membrane of a single cell was corrected for the background fluorescence, averaged and normalized for the background-subtracted ECFP fluorescence intensity measured in the cytosol of the same cell, to obtain wild type or mutant SLC26A4 expression levels normalized for the transfection efficiency of the single cell. Imaging was performed by confocal microscopy as described above. EYFP was excited with the 514 nm line of the Argon laser and emission was detected between 525 and 580 nm. ECFP was excited with a diode laser (405 nm) and emission was detected between 450 and 490 nm. CellMask^TM^ Deep Red stain was excited at 633 nm (HeNe laser) and emission was detected in the 643–750 nm range. Laser power and photomultipliers gain were kept rigorously constant for acquisition of all images.

### 4.10. Sequence Variation Genotyping Assays

Ten μL end-point genotyping reactions containing 1× TaqMan^®^ Universal PCR Master Mix no AmpErase^®^ UNG, 1× hydrolysis probe assay and 13.5 ng gDNA were performed in MicroAmp^®^ Optical 384-well Reaction Plates on the QuantStudio™ 12K Flex Real-Time PCR System (all from Thermo Fisher Scientific). Cycling parameters were: 60 °C for 30 s, 95 °C for 10 min., followed by 50 cycles of 92 °C for 15 s and 60 °C for 90 s, and a final 30 s at 60 °C. The two hydrolysis probe assays used in the 384-well format targeted *SLC26A4 c.343T>G* and *c.1301C>A* and were custom designed and manufactured by Thermo Fisher Scientific. 

### 4.11. Salts and Chemicals 

All salts and chemicals used were of pro analysis grade.

### 4.12. Statistical Analysis

All data are expressed as arithmetic means ± SEM and analyzed with GraphPad Prism software (version 4.00 for Windows, GraphPad Software, San Diego, CA, USA). Significant differences between data sets were verified by one way Analysis of Variance (ANOVA) with Bonferroni’s or Dunnet’s post-tests, or by the Fisher exact test, as appropriate. A *p* value < 0.05 was considered as statistically significant; (*n*) corresponds to the number of independent measurements. To determine departure from Hardy-Weinberg equilibrium, a chi-squared test with one degree of freedom was used.

## 5. Conclusions

To conclude, we presented the first characterization of a cohort of Austrian deaf patients with EVA. Biallelic *GJB2* and *SLC26A4* sequence variations with functional impact could be identified as the genetic determinant of deafness for two and one patient, respectively. Prevalence and spectrum of *SLC26A4* pathogenic variants are distinct from other Caucasian cohorts. Specifically, *SLC26A4* pathogenic variants seem to be under-represented in this cohort, thus providing an imperative towards the investigation of additional genes and/or genomic regions that may be involved in determining deafness and EVA. High throughput sequencing technologies, such as whole exome sequencing, would be of great value in this context. Further, this study underscores the importance of integrating genetic analysis, functional testing and identification of molecular features of protein variants to unambiguously identify or exclude the genetic cause of deafness.

## Figures and Tables

**Table 1:** Clinical signs of patients.

Patient ID	Ethnicity	Sex	Age (year)	EVA, Side	Mondini Malformation, Side	Side Affected by HL	Type of HL	Frequencies Affected by HL, Side
119	Caucasian	Male	33	B	No	B	Sensorineural	All, B
267	Caucasian (Arab)	Female	23	B	No	B	Sensorineural	All, B
271	Caucasian	Female	8	B	Yes, B	B	Sensorineural	All, B
272	Caucasian	Male	37	R	No	R	Conductive	Low and Middle, R
278	Caucasian	Female	42	L	No	B	Sensorineural	All, B
305	Caucasian	Male	16	B	Yes, B	B	Sensorineural	Middle and High, R; All, L
307	Caucasian	Male	18	B	No	B	Sensorineural	All, B
308	Caucasian (Arab)	Male	12	B	No	B	Sensorineural	All, B
358	Caucasian	Female	32	B	Yes, B	B	Sensorineural	All, B
359	Caucasian	Female	46	B	No	B	Sensorineural	All, R; Middle and High, L
365	Caucasian	Male	76	A	No	B	Sensorineural	All, B
395	Caucasian + African	Female	17	L	No	B	Sensorineural	Middle, R; All, L
421	Caucasian	Male	21	B	No	B	Sensorineural	All, B
568	Caucasian	Female	19	B	No	B	Sensorineural	All, B
569	Caucasian	Male	28	B	Yes, B	B	Sensorineural	All, B
610	Caucasian	Female	51	B	No	B	Sensorineural	All, B
616	Caucasian	Female	32	B	No	B	Mixed	Middle and High, R; Low and Middle, L
622	Caucasian	Male	10	B	Yes, B	B	Sensorineural	All, B
632	Caucasian	Female	50	B	No	B	Sensorineural	All, B

A, agenesis; B, bilateral; EVA, enlarged vestibular aqueduct; HL, hearing loss; L, left; R, right.

**Table 2:** *GJB2* and *SLC26A4* genotype of patients.

Patient ID	*GJB2*				*SLC26A4*				Causative Gene
	Nucleotide Change		Amino Acid Change		Nucleotide Change		Amino Acid Change		
	Allele 1	Allele 2	Allele 1	Allele 2	Allele 1	Allele 2	Allele 1	Allele 2	
119	*WT*	*WT*	WT	WT	*WT*	*WT*	WT	WT	undetermined
267	*c.88A>G*	*WT*	p.I30V	WT	*WT*	*WT*	WT	WT	undetermined
271	*WT*	*WT*	WT	WT	*c.1301C>A*	*c.1730T>C*	p.A434D	p.V577A	*SLC26A4*
272	*WT*	*WT*	WT	WT	*WT*	*WT*	WT	WT	undetermined
278	*WT*	*WT*	WT	WT	*WT*	*WT*	WT	WT	undetermined
305	*WT*	*WT*	WT	WT	*WT*	*WT*	WT	WT	undetermined
307	*c.35delG*	*c.35delG*	p.G12VfsX13	p.G12VfsX13	*WT*	*WT*	WT	WT	*GJB2*
308	*c.88A>G*	*WT*	p.I30V	WT	*WT*	*WT*	WT	WT	undetermined
358	*WT*	*WT*	WT	WT	*c.61A>G*	*WT*	p.M21V	WT	not *SLC26A4*
359	*WT*	*WT*	WT	WT	*WT*	*WT*	WT	WT	undetermined
365	*WT*	*WT*	WT	WT	*WT*	*WT*	WT	WT	undetermined
395	*WT*	*WT*	WT	WT	*WT*	*WT*	WT	WT	undetermined
421	*c.35delG*	*c.35delG*	p.G12VfsX13	p.G12VfsX13	*WT*	*WT*	WT	WT	*GJB2*
568	*WT*	*WT*	WT	WT	*WT*	*WT*	WT	WT	undetermined
569	*WT*	*WT*	WT	WT	*WT*	*WT*	WT	WT	undetermined
610	*WT*	*WT*	WT	WT	*WT*	*WT*	WT	WT	undetermined
616	*WT*	*WT*	WT	WT	***c.343T>G***	*WT*	**p.Y115D**	WT	undetermined
622	*WT*	*WT*	WT	WT	*WT*	*WT*	WT	WT	undetermined
632	*WT*	*WT*	WT	WT	*WT*	*WT*	WT	WT	undetermined

The novel pendrin sequence variant is indicated boldface. WT, wild type.

**Table 3:** Functional and molecular features of SLC26A4 variants.

SLC26A4 Variant	Function	Subcellular Localization	Expression Levels	Classification
p.M21V	not affected	plasma membrane	not affected	benign
p.Y115D	33% reduction	ER	reduced	pathogenic
p.A434D	16% reduction	plasma membrane and ER	reduced	pathogenic
p.V577A	76% reduction	ER	reduced	pathogenic

The % of reduction in function refers to the wild type, and was calculated by subtracting the intracellular fluorescence decrease observed in control cells from that observed in cells expressing each SLC26A4 variant. A classification of each variant according to the American College of Medical Genetics and Genomics Standards and Guidelines [26] based on functional and molecular features is given, and refers to a condition where a given variant is found in compound heterozygosity with another pathogenic variant. ER, endoplasmic reticulum.

## Supplementary Materials

Supplementary materials can be found at www.mdpi.com/1422-0067/19/1/209/s1.

## Abbreviations

ARNSHL	Autosomal Recessive Non-Syndromic Hearing Loss
ATP6V1B1	ATPase H^+^ Transporting V1 Subunit B1
CT	Computed Tomography
DFNB4	Deafness B4
ECFP	Enhanced Cyan Fluorescent Protein
ER	Endoplasmic Reticulum
EVA	Enlarged Vestibular Aqueduct
EYFP	Enhanced Yellow Fluorescent Protein
FOXI1	Forkhead Box I1
GJB2	Gap Junction Protein β2
HBSS	Hank’s Balanced Salt Solution
IP2	Incomplete Partition 2
KCNJ10	Potassium Channel, Inwardly Rectifying, Subfamily J, Member 10
MAF	Minor Allele Frequency
PS	Pendred Syndrome
RTA	Renal Tubular Acidosis
SLC26A4	Solute Carrier Family 26 Member A4
SNP	Single Nucleotide Polymorphism
