# Supplementary material for: Functional Testing of SLC26A4 Variants—Clinical and Molecular Analysis of a Cohort with Enlarged Vestibular Aqueduct from Austria
Source: Int J Mol Sci. 2018 Jan 10;19(1):209. doi: 10.3390/ijms19010209 (PMC5796158; doi:10.3390/ijms19010209)
Supplement: Supplementary file 1 [file ijms-19-00209-s001.pdf]

Supplementary Materials: Functional Testing of SLC26A4 Variants— Clinical and Molecular Analysis of a Cohort with Enlarged Vestibular Aqueduct from Austria

Sebastian Roesch, Emanuele Bernardinelli, Charity Nofziger, Miklós Tóth, Wolfgang Patsch, Gerd Rasp, Markus Paulmichl and Silvia Dossena

Table S1. Additional clinical features of patients regarding HL. B, bilateral; EVA, enlarged vestibular aqueduct; HL, hearing loss; L, left side; R, right side. n.a., not applicable.

Patient ID	Severity of HL, Side	Onset of HL, Side	Clinical course of HL	Hearing drops	Cochlear Implant, Side (age at implantation)	Intraoperative Gusher, Side	Family history of HL
119	Deaf, R<br>Severe to Profound, L	Postlingual, B	Progressive, R<br>Stable, L	No	R (24 years)	No	None
267	Profound, B	Postlingual, B	Progressive, B	No	L (18 years)	No	1 brother (patient ID 308) affected
271	Profound, R<br>Moderate, L	Congenital, B	Stable, R<br>Progressive, L	No	R (3 years),<br>L (5 years)	Yes, B	None
272	Mild, R	Postlingual, R	Stable, R	Yes, R, after trauma	No	n.a.	None
278	Mild, R<br>Profound, L	Postlingual, R<br>Congenital, L	Progressive, R<br>Stable, L	No	L (37 years)	No	None
305	Mild to Moderate, R<br>Deaf, L	Postlingual, B	Stable, R<br>Progressive L	Yes, L, age of 13	No	n.a.	Yes
307	Deaf, B	Congenital, B	Stable, B	No	R (14 years),<br>L (3 years)	No	None
308	Profound, R<br>Severe, L	Postlingual, B	Stable, R<br>Progressive, L	No	R (10 years)	Yes, R	1 sister (patient ID 267) affected
358	Deaf, R<br>Profound, L	Congenital, R<br>Postlingual, L	Stable, R<br>Progressive, L	Yes, L, age of 23	L (25 years)	No	Yes
359	Deaf, R<br>Profound, L	Congenital, R<br>Postlingual, L	Stable, R<br>Progressive, L	Yes, L, age of 29	R (42 years),<br>L (44 years)	No	1 aunt (mother side) congenitally deaf
365	Severe, R<br>Deaf, L	Postlingual, B	Progressive, R<br>Stable, L	Yes, B;<br>L age of 18, R age of 59	L (68 years) - explant (71 years)	No	None
395	Moderate, R<br>Deaf, L	Postlingual, B	Stable, B	No	L (15 years)	No	None
421	Severe, R<br>Profound, L	Perilingual, B	Stable, B	No	L (20 years)	No	None

568	Severe, B	Perilingual, B	Stable, B	No	R (16 years),<br>L (6 years)	No	None
569	Severe, B	Congenital, B	Progressive, B	Yes, B, age of 6 and 13	R (17 years),<br>L (7 years/24 years)	Yes, B	None
610	Profound, B	Postlingual, B	Progressive, B	Yes, B;<br>L age of 27 after birth of daughter	R (50 years)	No	4 generations affected
616	Mild to profound, R<br>Mild to moderate, L	Perilingual, B	Fluctuating, R<br>Stable, L	No	No	n.a.	None
622	Profound, B	Congenital, B	Stable, B	No	R (2 years),<br>L (3 years)	Yes, B	None
632	Profound, B	Postlingual, B	Progressive, B	No	R (42 years),<br>L (42 years)	No	1 sister congenitally deaf

Table S2. Clinical features of patients regarding the vestibular phenotype and history of migraine. B, bilateral; L, left side; R, right side; SCC, semicircular canal; n.a., not assessed.

Patient ID	Subjective Vertigo	Problems in learning to walk	Vestibular function		Migraine
			Caloric testing - slow phase velocity of nystagmus	Video Head Impulse Test	
119	Episodic	None	Physiological, L; Reduced, R	n.a.	No
267	None	None	n.a.	n.a.	No
271	None	None	n.a.	n.a.	No
272	Episodic	None	Reduced, B	Pathological, R - Anterior and Horizontal SCC; Pathological, L - Anterior SCC	No
278	Episodic	Prolonged	Reduced, L; Physiological, R	n.a.	No
305	None	None	n.a.	Physiological, B	No
307	Episodic	None	n.a.	n.a.	No
308	None	None	n.a.	n.a.	No
358	Episodic	None	Reduced, B	n.a.	No
359	Episodic	None	Physiological, L; n.a., R	Physiological, B	No
365	Progressive	None	Reduced, B	n.a.	No
395	Episodic	Prolonged	Reduced, B	n.a.	Headache [1]
421	Episodic	Prolonged	Physiological, L; Reduced, R	n.a.	No
568	Episodic	None	n.a.	n.a.	No
569	None	None	n.a.	n.a.	No
610	Episodic	None	Physiological, B	Physiological, B	No
616	Episodic	None	Physiological, B	Physiological, B	No
622	None	None	n.a.	n.a.	No
632	None	None	Physiological, B	n.a.	No

[1] With no characteristics of migraine.

Table S3. All *SLC26A4* sequence variations detected by Sanger sequencing. The gDNA and cDNA positions are according to reference sequences AC078937.1 and NM_000441, respectively. The translation start site in the following context sequence is underlined: 5' <u>ATG</u>GCAGCGCCAGGCGGCAGGTCGGAGC 3'. The "A" of the ATG denotes +1 and the nucleotide just before denotes -1. Unfilled boxes refer to the wild-type nucleotide, whereas the grey and black boxes indicate that the patient is heterozygous or homozygous mutant for the variation shown, respectively. **There is a dbSNP ID for this position (rs564434827), but it is described as -431C>T. *rs56017519 is reported as tri-allelic (39682T>C or G). For some samples, particular positions were not covered by the sequencing reaction, and are denoted as n/a.

		UpstreamRegion		Exon 1	Intron 1		Exon 2	Intron 2		Exon 4	Intron 4			Intron 5	Intron 8	Intron 9	Exon 11	Intron 12	Intron 13	Intron 15	Exon 16	Intron 17					Intron 19	3'UTR			
	dbSNP ID (chromosomal position, GRCh38.p7)	rs2701685 (107659139)	rs2712228 (107659895)	rs17154282 (107660793)	** (107661211)	rs55753070 (107661378)	rs375716219 (107661702)	rs2248464 (107663177)	rs2248465 (107663183)	none (107672176)	rs11769313 (107673502)	none (107673783)	none (107673856)	rs3817613 (107674679)	rs2395911 (107683668)	rs2072063 (107689989)	none (107694440)	rs56045331 (107694959)	rs73421452 (107696437)	rs55701254 (107701083)	rs56017519* (107701123)	rs982662 (107703509)	rs982663 (107703588)	rs982664 (107703771)	rs2188562 (107703772)	rs6955309 (107704121)	rs151163258 (107712082)	rs2712218 (107716314)	rs190337640 (107717235)	rs76820337 (107717243)	rs375630290 (107717351)
	gDNA position (cDNA position)	-2503A>G	-1747A>C	-849C>G	-431delC	-264C>T	61A>G (c.61A>G)	1536A>T	1542C>T	10535T>G (c.343T>G)	11861G>A	12142C>T	12215G>A	13038T>G	22027G>T	28548C>T	32999C>A (c.1301C>A)	33518T>C	34996T>G	39642T>A	39682T>C (c.1730T>C)	42068T>A	42147A>G	42330C>T	42331T>C	42680C>T	50641A>G	54873G>A	55794G>A	55802G>C	55910G>A
	Amino Acid Change						M21V			Y115D							A434D				V577A										
Patient ID	119							n/a	n/a		n/a	n/a	n/a	n/a		n/a		n/a	n/a			n/a	n/a	n/a	n/a	n/a	n/a				
	267	n/a	n/a																												
	271	n/a	n/a																												
	272	n/a	n/a																												
	278	n/a	n/a																												
	305	n/a	n/a																												
	307	n/a	n/a												n/a																
	308	n/a	n/a																												
	358	n/a	n/a																												
	359	n/a	n/a												n/a																
	365	n/a	n/a																												
	395	n/a	n/a																												
	421	n/a	n/a																												
	568	n/a	n/a																												
	569	n/a	n/a																												
	610	n/a																													
	616	n/a	n/a																												
	622	n/a	n/a																												
	632	n/a	n/a																												

Table S4. Minor allele frequency (MAF) of the indicated *SLC26A4* variants. The symbol "-" denotes that the corresponding variant was not reported in a given database. Databases were accessed on December 2017.

Nucleotide change	Amino acid change	MAF			
		SNPdb [1]	ClinVar [2]	GnomAD [3]	DVD [4]
c.61A>G	p.M21V	0.00004203	-	0.0001939	0.0001805
c.343T>G	p.Y115D	-	-	-	-
c.1301C>A	p.A434D	-	-	0.00003231	-
c.1730T>C	p.V577A	0.00001647	-	0.000004067	0.00001651

[1] https://www.ncbi.nlm.nih.gov/snp/
[2] https://www.ncbi.nlm.nih.gov/clinvar
[3] http://gnomad.broadinstitute.org/
[4] http://deafnessvariationdatabase.org/

Table S5. Specifications for *SLC26A4* (reference sequence AC078937.1) amplification. PCR cycling parameters were 94°C x 2 min, followed by 35 cycles of 94°C × 20 sec, the indicated Tm × 30 sec, and 68°C × the indicated extension time. Primers in bold face type were designed in house. All others were already reported [1].

***SLC26A4* Region**	**Size (bps)**	**PCR Primer Sequences**	**Tm (°C)**	**Extension Time (min)**	**Sequencing Primer Sequences**
**5' UTR–Exon 3**	4447	**5' AAAGGAGACACAGTGCTTGCCCTC 3'** (fwd)<br>5' GAAGGGTAAGCAACCATCTGTCAC 3' (rev)	59.6	5.50	**5' GTCCCCTTCCAGCCTTGCAAGCGCCTTTGG 3'** (fwd)<br>**5' CTCCCAGAGACCACGGACCTCTTC 3'** (fwd)<br>5' CTTTCATCTGTAGGTCACTG 3' (fwd)<br>**5' CCAAAGGCGCTTGCAAGGCTGGAAGGGGAC 3'** (rev)<br>**5' CTCCGCCGCCCGCACCCCACTCTCGCCCGCTG 3'** (rev)<br>**5' GAAGGGTAAGCAACCATCTGTCAC 3'** (rev)
**Exons 4–6**	3049	5' TAATCACTTTGCATGTGCTTT 3 (fwd)<br>5' ATTGTTTCTGGAATGAACAGTGACC 3' (rev)	49.6	5.50	5' TAATCACTTTGCATGTGCTTT 3' (fwd)<br>5' CCTATGCAGACACATTGAACATTTG 3' (fwd)<br>5' GCCAAAACACTTTAAACATGA 3' (rev)<br>5' ACCTGTATAATTCCAACCAGCA 3' (rev)<br>5' ATTGTTTCTGGAATGAACAGTGACC 3' (rev)
**Exons 7–8**	636	5' CATGGTTTTTCATGTGGGAAGATTC 3' (fwd)<br>5' CAAATGGCTTGACGTTTATCTACACAC 3' (rev)	53.4	1.00	same as PCR primer sequences
**Exons 9–10**	1297	5' GTGGTCAAATCTTCACAGCA 3' (fwd)<br>5' CGAGCCTTCCTCTGTTGC 3' (rev)	53.4	2.00	5' GTGGTCAAATCTTCACAGCA 3' (fwd)<br>5' AAATACTCAGCGAAGGTCTTGC 3' (fwd)<br>5' CCCTTCTTTAGCTGACACCA 3' (rev)<br>5' CGAGCCTTCCTCTGTTGC 3' (rev)
**Exons 11–14**	3895	5' GGGGAGACAGGGAAGTATGAAGTG 3' (fwd)<br>5' AATGGAGCTGCTGAAACTTC 3' (rev)	51	5.50	5' GGGGAGACAGGGAAGTATGAAGTG 3' (fwd)<br>5' TTGTTTGTGGATCATTGATCTT 3' (fwd)<br>**5' CCACACAAACACCAGC 3'** (fwd)<br>5' AATGGAGCTGCTGAAACTTC 3' (rev)<br>**5' CCACAATTCTAATTTCTCCTCTGG 3'** (rev)<br>**5' CGCCTTACATTCCTCATCTC 3'** (rev)
**Exons 14–16**	3272	5' CAAAATACGGCTGTTCCAAA 3' (fwd)<br>5' GACCCTCTAACTGCTCTCATCA 3' (rev)	51	5.50	**5' CTGAGCAACTGTGACTTGAC 3'** (fwd)<br>**5' CACAAAGCCTGTTAGTCCAAC 3'** (rev)
**Exons 16–18**	3432	5' AAATACTCAGCGAAGGTCTTGC 3' (fwd)<br>5' GATAGGAGAAAGGGCTTACGG 3' (rev)	51	5.50	**5' CTTGAACAGAGGTCTTGATTTG 3'** (fwd)<br>5' TTGAGAAATAGCCTTTCCAGAT 3' (fwd)<br>5' CCCATGTATTTGCCCTGTTGC 3' (rev)<br>5' GATAGGAGAAAGGGCTTACGG 3' (rev)
**Exon 19**	409	5' GTTGCAGTGAGCAATGATGCC 3' (fwd)<br>5' CTGATGAAAAAACTGAGGCTC 3' (rev)	53	0.75	same as PCR primer sequences
**Exon 20**	718	**5' GAAGATTAAATTACATGCCACCTC 3'** (fwd)<br>5' GCATTTGGGGGAATTATGTT 3' (rev)	51.3	1.00	same as PCR primer sequences
**Exon 21–3'UTR**	2446	5' ACACTTTGTTTTCCCCTTGC 3' (fwd)<br>**5' CTCCTGGCACATTCTTTATTATATTTAATAG 3'** (rev)	49.6	5.50	**5' GAACCAGGCCAATATATTTTG 3'** (fwd)<br>**5' CTTGGTATACTCCAGGGATTG 3'** (fwd)<br>**5' ATCTTTAGGCAGGGGTGAC 3'** (fwd)<br>**5' GGCCTGGTTCTGTAGCTTTTAG 3'** (fwd)<br>5' ACACTTTGTTTTCCCCTTGC 3' (fwd)<br>**5' GAGTGAATGTAATAGTCTTGCAGAAAATG 3'** (fwd)<br>**5' CTCCTGGCACATTCTTTATTATATTTAATAG 3'** (rev)

Table S6. Mutagenesis primers used to obtain *SLC26A4* variants. The nucleotide variant is underlined.

***SLC26A4* variant**	**forward**	**reverse**
***c.61A>G* (p.M21V)**	5′ TAC AGC TGC AGC TAC GTG GTG TCG CGG CCG G 3′	5′ CCG GCC GCG ACA CCA CGT AGC TGC AGC TGT A 3′
***c.343T>G* (p.Y115D)**	5′ GCA GTT CCT GTC GGA GAT GGT CTC TAC TCT GC 3′	5′ GCA GAG TAG AGA CCA TCT CCG ACA GGA ACT GC 3′
***c.1301C>A* (p.A434D)**	5′ CGA TTG TGA TGA TCG ACA TTC TTG CCC TGG GG 3′	5′ CCC CAG GGC AAG AAT GTC GAT CAT CAC AAT CG 3′
***c.1730T>C* (p.V577A)**	5′ TTG ATG CCA TTA GAG CAT ATA ATA AGA GGC TG 3′	5′ CAG CCT CTT ATT ATA TGC TCT AAT GGC ATC AA 3′

References

1. Everett, L.A.; Glaser, B.; Beck, J.C.; Idol, J.R.; Buchs, A.; Heyman, M.; Adawi, F.; Hazani, E.; Nassir, E.; Baxevanis, A.D.; Sheffield, V.C.; Green, E.D. Pendred syndrome is caused by mutations in a putative sulphate transporter gene (*PDS*). *Nat. Genet.* **1997,** *17*, 411–422, Doi 10.1038/Ng1297-411.

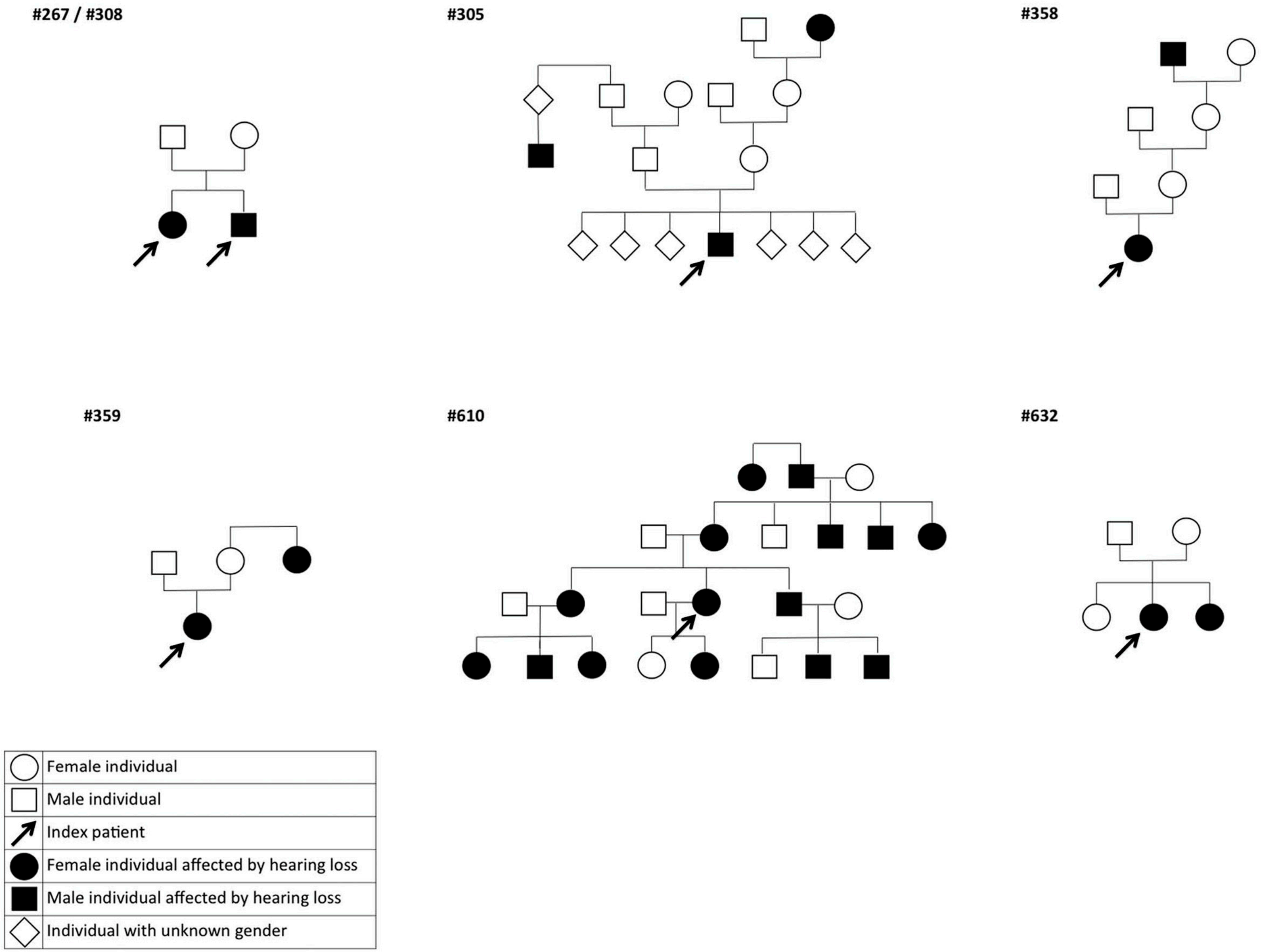

Figure S1. Pedegree of families with a history of hearing loss. The patient ID indicated refers to the index patient.
